# Transcriptome Sequencing of Gene Expression in the Brain of the HIV-1 Transgenic Rat

**DOI:** 10.1371/journal.pone.0059582

**Published:** 2013-03-25

**Authors:** Ming D. Li, Junran Cao, Shaolin Wang, Ju Wang, Sraboni Sarkar, Michael Vigorito, Jennie Z. Ma, Sulie L. Chang

**Affiliations:** 1 Department of Psychiatry and Neurobehavioral Sciences, University of Virginia, Charlottesville, Virginia, United States of America; 2 School of Biomedical Engineering, Tianjin Medical University, Tianjin, China; 3 Institute of NeuroImmune Pharmacology, Seton Hall University, South Orange, New Jersey, United States of America; 4 Department of Biological Sciences, Seton Hall University, South Orange, New Jersey, United States of America; 5 Department of Psychology, Seton Hall University, South Orange, New Jersey, United States of America; 6 Department of Public Health Sciences, University of Virginia, Charlottesville, Virginia, United States of America; Temple University School of Medicine, United States of America

## Abstract

The noninfectious HIV-1 transgenic (HIV-1Tg) rat was developed as a model of AIDs-related pathology and immune dysfunction by manipulation of a noninfectious HIV-1^gag-pol^ virus with a deleted 3-kb *Sph*I*-Msc*I fragment containing the 3′ -region of *gag* and the 5′ region of *pol* into F344 rats. Our previous studies revealed significant behavioral differences between HIV-1Tg and F344 control rats in their performance in the Morris water maze and responses to psychostimulants. However, the molecular mechanisms underlying these behavioral differences remain largely unknown. The primary goal of this study was to identify differentially expressed genes and enriched pathways affected by the *gag-pol*-deleted HIV-1 genome. Using RNA deep sequencing, we sequenced RNA transcripts in the prefrontal cortex, hippocampus, and striatum of HIV-1Tg and F344 rats. A total of 72 RNA samples were analyzed (i.e., 12 animals per group × 2 strains × 3 brain regions). Following deep-sequencing analysis of 50-bp paired-end reads of RNA-Seq, we used Bowtie/Tophat/Cufflinks suites to align these reads into transcripts based on the Rn4 rat reference genome and to measure the relative abundance of each transcript. Statistical analyses on each brain region in the two strains revealed that immune response- and neurotransmission-related pathways were altered in the HIV-1Tg rats, with brain region differences. Other neuronal survival-related pathways, including those encoding myelin proteins, growth factors, and translation regulators, were altered in the HIV-1Tg rats in a brain region-dependent manner. This study is the first deep-sequencing analysis of RNA transcripts associated the HIV-1Tg rat. Considering the functions of the pathways and brain regions examined in this study, our findings of abnormal gene expression patterns in HIV-1Tg rats suggest mechanisms underlying the deficits in learning and memory and vulnerability to drug addiction and other psychiatric disorders observed in HIV-positive patients.

## Introduction

Human immunodeficiency virus-1 (HIV-1) infection involves the actions of HIV-1 viral proteins on targeted cells of the immune system, such as macrophages and T-lymphocytes [1]–[6]. Like the peripheral immune system, the central nervous system (CNS) is highly vulnerable to HIV-1 infection. The virus targets several brain regions, including the striatum (STR), prefrontal cortex (PFC), and hippocampus (HIP) [7], [8], which regulate learning, memory, and motivation [9]–[12]. The neuropathology resulting from HIV-1 infection is characterized by progressive cognitive decline, motor dysfunction, striatal pathology, and gliosis [13], [14]. Although highly active anti-retroviral therapy (HAART) leads to a temporary reversal of HIV dementia [15]–[17], both the HIV-associated neurocognitive disorders (HAND) and HIV-associated dementia (HAD) are resistant to HAART and become more severe as the lifespan of HIV-1-infected patients is prolonged [18]. Thus, a thorough understanding of how HIV-1 infection induces cognitive dysfunction is greatly needed. Given that HAART can inhibit viral entry and replication without eliminating the viral proteins [19]–[23], the clinical challenge in this post-HAART era is to determine the effects of the persistent presence of HIV viral proteins in the host [20], [24], [25].

To better understand the effect of HIV-1 on neurocognitive function, an HIV-1 transgenic (HIV-1Tg) rat model, which carries a *gag-pol*-deleted HIV-1 genome under the control of the HIV-1 viral promoter and expresses 7 of the 9 HIV-1 genes, was developed [26]. Although there is no viral replication, viral proteins are expressed in various organs [26] and are found in the blood [26], [27]. The HIV-1Tg rats develop characteristics similar to those of humans infected with HIV-1, including immune-response alterations, pathologies with advancing age [26], T-cell abnormalities [28], and kidney failure [29]. Neurobehavioral and neuropathologic changes within the brain parenchyma of HIV-1Tg rats have been documented [26]. Similar to HIV-1-positive patients, HIV-1Tg rats exhibit deficits in learning and memory [30], [31]. The rats also show different sensitivity to many psychostimulants, including morphine, alcohol, and nicotine [32]–[34]. The development of various manifestations of human HIV-1 infection in the HIV-1Tg rat, without viral replication, indicates that it is the presence of viral proteins in the host that affects the targeted cells, including immune cells, and causes the clinical progression to AIDS. Thus, the HIV-1Tg rat appears to mimic the condition of HIV-1 patients given HAART, who have controlled viral replication, but persistent HIV-1 infection that is often associated with slow progressive neurodegeneration and eventually advances to AIDS.

Although a number of studies have been conducted on the HIV-1Tg rat, most of them focused on a limited number of genes. Given the complexity of the neuropathological changes associated with HIV-1 infection, it is of great interest to examine most, if not all, genes and biological cascades affected by HIV-1 in the HIV-1Tg rat using a high-throughput approach such as second-generation sequencing technology. Delineating the overall gene expression profile in the brain of the HIV-1Tg rat will help to identify the mechanisms involved in HIV-1 neuropathology and allow for the development of efficient therapy for cognitive deficits and other neuropsychiatric disorders associated with HIV-1 infection.

## Materials and Methods

### Animals

The Animal Care and Use Committee of both the Seton Hall University and University of Virginia approved this study. Adult male HIV-1Tg rats and F344 background control rats (n = 12 per group) were purchased from Harlan Inc. (Indianapolis, IN). All rats were double housed in standard plastic cages and maintained in a temperature-controlled environment with a 12 h light/dark cycle and fed a standard rat diet and water *ad libitum*. The animals were monitored daily, and their cage bedding was changed twice a week. All animals were participants in a previously reported behavioral study [31]. All experimental procedures were conducted during the light cycle in accordance with the Animal Care and Use Committees of both participating institutions.

### Tissue Collection

Using a rat brain matrix, slices of approximately 1 mm were taken from each brain, and the slices that contained the PFC, HIP, and dorsal STR were identified according to a rat brain atlas [35]. Tissues from specific regions of interest were collected bilaterally from each brain using a 3.00-mm Harris Micro-Punch (GE Healthcare Life Sciences, Piscataway, NJ, USA) and stored at −80°C until use.

### RNA Extraction and Sample Preparation

Total RNA was extracted from each tissue sample using TRIzol (Life Technologies, Grand Island, NY) according to the protocol provided by the manufacturer. The RNA concentration of each sample was quantified using the Qubit RNA BR Assay Kit (Life Technologies, Grand Island, NY) and the quality was assessed using the Agilent Bioanalyzer 2100 (Agilent, Santa Clara, CA).

### RNA Sequencing Library Preparation and Deep Sequencing

The sequencing library of each RNA sample was prepared with the TruSeq RNA Sample Preparation kit based on the protocol provided by the manufacturer (Illumina, San Diego, CA). Briefly, poly(A)-containing mRNA was purified from 1 µg of RNA with streptavidin-coated magnetic beads. After chemical fragmentation, mRNA fragments were reverse-transcribed and converted into double-stranded cDNA. Following end repair and A-tailing, paired-end sequencing adaptors complementary to sequencing primers were ligated to the ends of the DNA fragments. The ligated products were purified on 2% agarose gels, and 200–250-bp fragments were selected for downstream enrichment by 15 cycles of PCR followed by purification using a QIAquick PCR purification kit (Qiagen). The enriched libraries were diluted with elution buffer to a final concentration of 10 nM. Each sample (ca. 7 pM concentration) was subjected to 50 cycles of sequencing from both ends in one lane of an Illmina Hiseq2000 Sequencer.

### Pre-processing and Mapping of RNA-seq Reads using TopHat

The extraction of 50-bp length paired-end reads was achieved using CASAVA (Illumina Pipeline v1.38). For each sample, reads with a quality score of ≥Q30 that passed filtering were used to generate a complete FASTQ file, which was then mapped to UCSC Rat reference [build Rn4] (ftp://ftp.cbcb.umd.edu/pub/data/bowtie_indexes/rn4.ebwt.zip) using TopHat with the default parameter setting of 40 alignments per read and up to 2 mismatches per alignment. The sequence alignment files (BAM) were analyzed using RSeQC package [36] for quality control analysis, which includes the mRNA fragment insert size, base quality distribution, reads mapping distribution, and splicing distribution analysis.

The resulting aligned reads were then analyzed with Cufflinks suite (http://cufflinks.cbcb.umd.edu) [37], which assembles the aligned reads into transcripts and measures their relative abundance. The expression of each transcript was quantified as the number of reads mapping to a gene divided by the gene length in kilobases and the total number of mapped reads in millions, which is called fragments per kilobase of exon per million fragments mapped (FPKM). All the junctions identified by Cufflink were compared on the basis of the junction and splicing site provided by reference transcript annotation GTF files to identify known and novel junctions. Then, Cuffcompare merged all the transcripts from different samples to a final transcript annotation GTF file, reported changes in the relative abundance of transcripts sharing a common transcription start site, and indicated the relative abundance of the primary transcripts of each gene crossing all the samples.

### Gene Annotation and Expression Profiling Analysis

The Ensembl transcript ID was used as the primary identifier for all our analyses. When multiple splice variants existed, all of them were selected. In generating the RPKM distributions of intergenic regions, we considered regions with a distance of at least 10 kb from any RefSeq or Ensembl gene. The annotation information corresponding to each Ensembl transcript ID was retrieved from the Ensembl database via BioMart (http://www.biomart.org/biomart/martview). To convert the Ensembl Transcript ID to Gene ID, we selected “Ensembl gene 66″ for the database and “Rattus norvegicus genes” for the dataset. Together, information on 39,550 unique transcripts was retrieved, and 22,920 of them were assigned to function-defined protein-coding genes, whereas the remaining transcripts corresponded to predicted genes or different types of RNAs.

For each brain region of interest, all the transcripts were pulled from the file generated by Cufflinks. The measurements with RPKM values close to zero (about 5% of the total) were discarded. The RPKM values were logarithmically transformed to base 2, and the measurements of each transcript within an experimental group were subjected to outlier detection [38]. Transcripts with fewer than six valid measurements in either comparison group after the removal of outliers were discarded. According to the number of transcripts mapped to a gene, the following three cases were considered: 1) where a single transcript was mapped to a gene, we used the corresponding intensities in all further analysis; 2) where there were multiple records for a single transcript in the dataset (≥6 in each group of 12 samples), the intensity values were averaged and treated as one record; and 3) where multiple transcripts mapped to the same gene, they were treated as independent genes in the data analysis steps.

A Student’s *t*-test was used to identify differentially expressed genes in each brain region in the two animal strains. On the basis of the *p* values, the false discovery rate (FDR) was calculated by the method of Benjamini and Hochberg [39]. All analyses were conducted with MATLAB (The Mathworks Inc., Natick, MA). Reported significance was defined as p<0.005 (FDR <0.20) with a fold change (FC) >20%. Genes with 0.005<p<0.05 were considered as having marginal significance, as documented in Table S1.

### Enriched Biochemical Pathways in the HIV-1Tg Rat

The genes significantly altered in the HIV-1Tg rat were analyzed by Ingenuity Pathway Analysis (IPA; https://analysis.ingenuity.com), with the goal of revealing the enriched biochemical pathways. The core component of IPA is the Ingenuity Pathways Knowledge Base (IPKB), which contains the biological function, interaction, and related information of a curated gene set and more than 330 biochemical pathways. This pathway-based software is designed to identify global canonical pathways, dynamically generated biological networks, and global functions from a given list of genes. Basically, the genes with their symbols, corresponding GenBank accession numbers, or both were uploaded into the IPA and compared with the genes included in each canonical pathway using the whole gene set of IPKB as the background. All the pathways with one or more genes overlapping the candidate genes were extracted. In IPA, each of these pathways was assigned a *p* value via Fisher’s exact test, which denoted the probability of overlap between the pathway and input genes.

### Quantitative Real Time RT-PCR

To further confirm the findings from the RNA-seq analysis, we conducted quantitative real-time RT-PCR (qRT-PCR), the traditional quantification method on gene expression. We selectively examined interferon regulatory factor 7 (*Irf7*) and Nucleophosmin (*Npm1*) with the same RNA samples used for RNA-seq analysis, considering they are the only genes showing strain differences in all the three brain regions based on the deep sequencing analysis results. Primers used in the qRT–PCR array were designed using Primer Express (v. 3.0; Applied Biosystem Inc., CA, USA) software. The selected primer sequences were further subjected to a BLAST search to ensure specificity of the primers for the target gene and were synthesized by Fisher Scientific (USA). The primer sequences are listed below: *Irf7* forward primer: 5′-TTGCTTCAGGTTCTGCAATACAG-3′, *Irf7* reverse primer: 5′-TCCTCAGGAAGGTGTTCTTGCT-3′, *Npm1* forward primer: 5′-CTATGAAGGCAGCCCAATTAAAG-3′, and *Npm1* reverse primer: 5′-CCACAGGTGGTGTAATTTCGAA-3′. The qRT-PCR analysis was done as described previously [40]–[43]. Briefly, RT product was amplified in a volume of 10 µl containing 5 µl 2× Power SYBR® Green PCR Master Mix (Applied Biosystems, CA, USA), and combined sense and antisense primers (2.5 µl, final concentration 250 nm) and 2.5 ul diluted cDNA in a 384-well plate using the 7900HT Fast Real Time PCR system (Applied Biosystems). Expressions of all genes were normalized to the expression of housekeeping gene hydroxymethylbilane synthase (*Hmbs*) and then analyzed using a comparative *C_t_* method [44]. *Hmbs* was identified by Normfinder program [45] as the most stably expressed housekeeping gene between the two strains. The relative gene expression was compared between F344 and HIV-1Tg rats using the Student’s *t* test with the Bonferroni adjustment for multiple comparisons (*N* = 6 per group).

## Results

### Overview and Summary of Sequencing Data from RNA-seq Analysis

**Figure 1:**
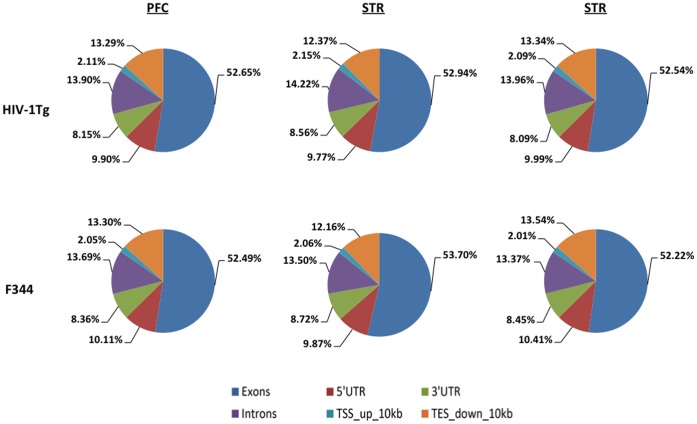
Percentage of mapped reads onto the regions of exons, introns, 5′ -UTRs, 3′ -UTRs, and 10-kb transcribed region upstream and downstream from coding regions in the PFC, STR, and HIP of HIV-1Tg and F344 rats.

Total raw data among samples ranged from 45 to 65 million reads, with an average of approximately 54 million raw reads per sample. More than 95% bases had a quality score of ≥ Q30, and the mean quality score was Q37. The raw sequencing data averaged about 2.5 GB of data (50 × coverage of transcriptome) per sample. About 40∼55 × 10^6^ reads (77–83% of the total raw reads) were uniquely aligned to Rat genome sequence (Build rn3.4) among samples (Table 1), with an average of 44 × 10^6^ reads per sample. Sequencing and mapping quality were analyzed using RSeQC, which showed that the average mRNA insert fragment size was 159 bp (±52 bp; SD). The distribution of mapped reads on the genome was consistent among samples (Fig. 1). Of the uniquely mapped reads, more than 70% were aligned at the transcript exon and UTR regions, 14% at the intron regions, and the remaining 16% at within 10 kb upstream and downstream of the transcript (Fig. 1).

**Table 1:** Statistics of raw and mapped reads from RNA-seq analysis in the PFC, STR, and HIP of HIV-1Tg and F344 rats.

Strain	PFC		STR		HIP	
	Raw Reads*	Mapped Reads*	Raw Reads*	Mapped Reads*	Raw Reads*	Mapped Reads*
F344	60.77±10.33	49.84±7.50	48.08±4.24	39.44±3.37	48.84±7.70	39.28±5.34
HIV-1Tg	65.56±10.27	54.27±8.15	56.94±4.08	44.34±4.47	46.45±2.25	38.49±1.87

* The unit used to measure the number of reads is million.

**Figure 2:**
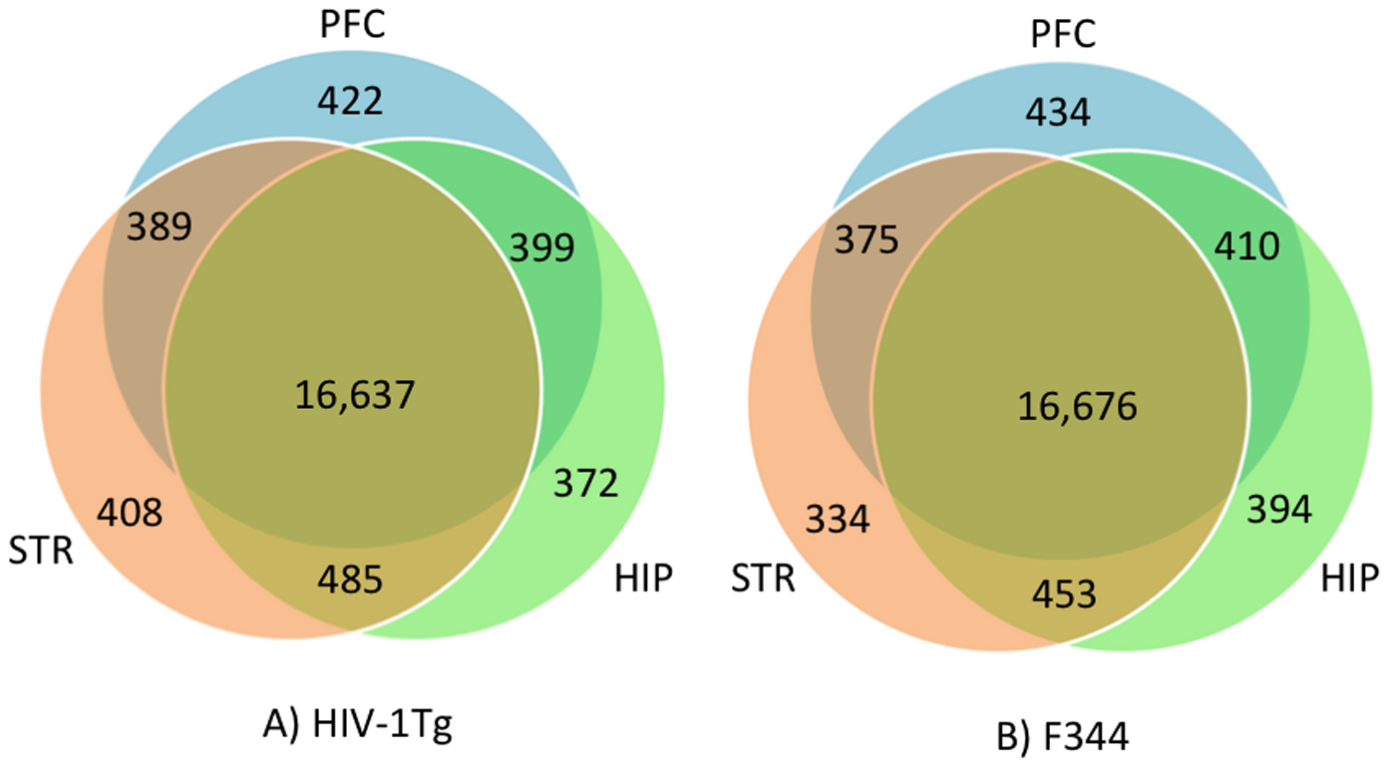
Comparison of the number of transcripts identified in the three brain regions in HIV-1Tg (A) and F344 (B) rats.

These aligned reads were subsequently analyzed by Cufflinks suites for transcript assembly, abundance evaluation, and normalization. An average of 50,071 transcripts among all samples was obtained. After annotation, there were 22,920 transcripts annotated with known function from the Ensembl database. For HIV-1Tg animals, we identified 17,893, 17,847, and 17,919 transcripts with known function in the HIP, PFC, and STR, respectively, with 16,637 common transcripts among the three brain regions (Fig. 2A). For F344 animals, 17,933, 17,895, and 17,838 transcripts with known function were identified in the HIP, PFC, and STR, respectively; a total of 16,676 common transcripts were identified among the three brain regions (Fig. 2B).

### Identification of Genes and Pathways Altered in the HIV-1Tg Rat

**Figure 3:**
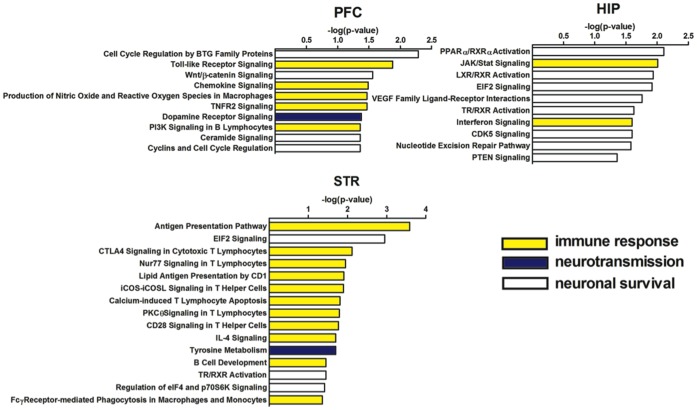
Enriched biochemical pathways significantly altered in HIV-1Tg rats compared to F344 control rats in the PFC, HIP, and STR. These pathways were divided into three main groups directly related to immune responses (yellow bars), neurotransmission (blue bars), and neuroplasticity (white bars).

We found 197, 154, and 171 differentially expressed genes in the PFC, HIP, and STR regions, respectively, in HIV-1Tg rats compared to F344 rats. To understand these changes at the pathway level, we conducted an IPA analysis and found 10, 10, and 15 signaling pathways to be significantly altered in the PFC, HIP, and STR regions, respectively, in the HIV-1Tg rat (Fig. 3). Most pathways were altered in only one brain region, except that EIF2 signaling and TR/RXR activation were altered in both the HIP and STR.

Reviewing the biological functions of each IPA-identified pathway, we found many were highly related, with four pathways in the PFC, two in the HIP, and eleven in the STR being related to immune responses. The affected neurotransmission-related pathways included dopamine receptor signaling, which was altered in the PFC, and tyrosine metabolism, which was affected in the STR. The remaining pathways are all related to neuronal survival, with five pathways in the PFC, eight in the HIP, and three in the STR.

**Figure 4:**
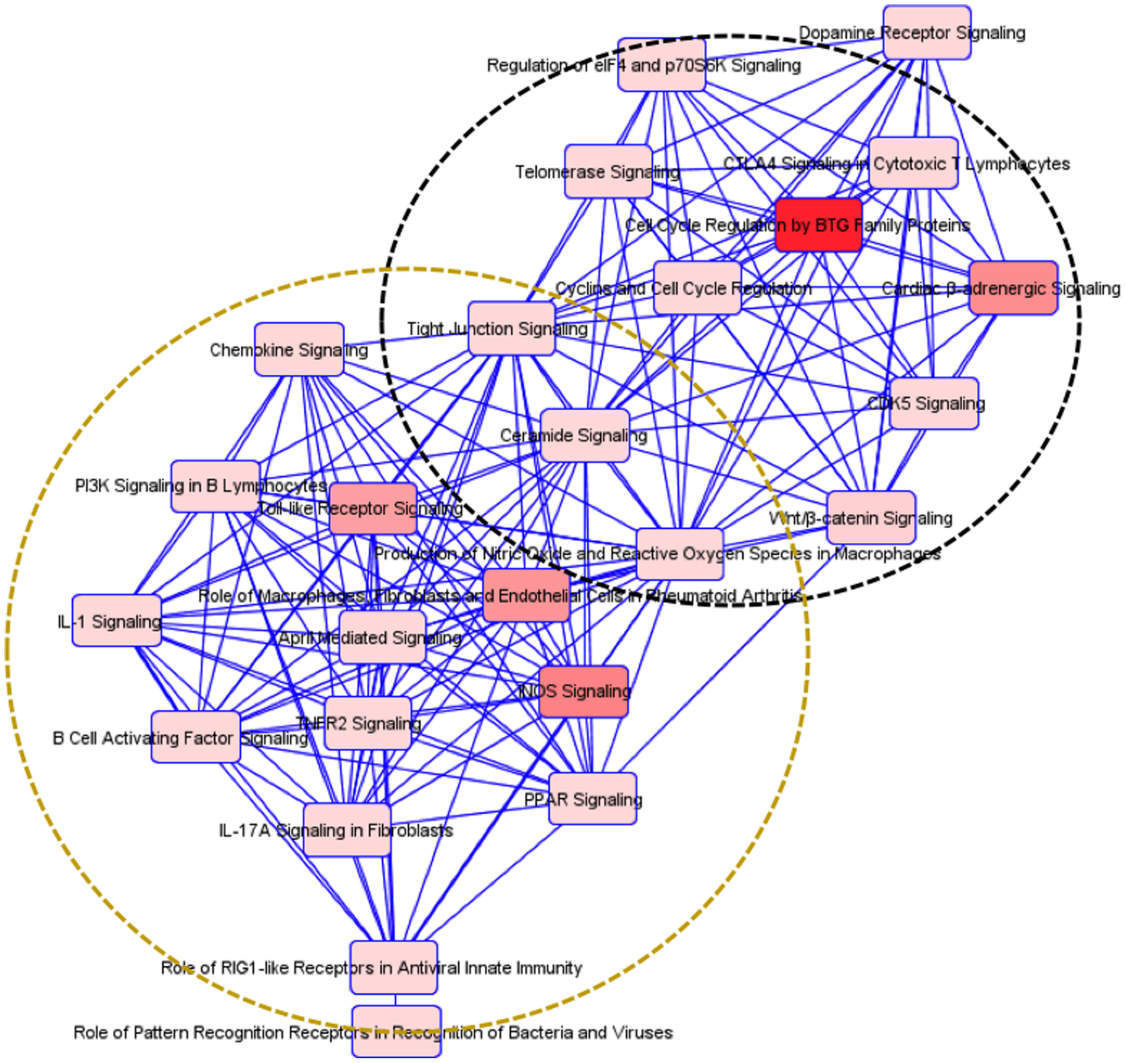
Detected interactions and clusters of enriched pathways in the PFC region in HIV-1Tg rats. All pathways identified by IPA software, including both significant and non-significant pathways, are highly interrelated and can be divided into two function-related groups, indicated by circles.

**Figure 5:**
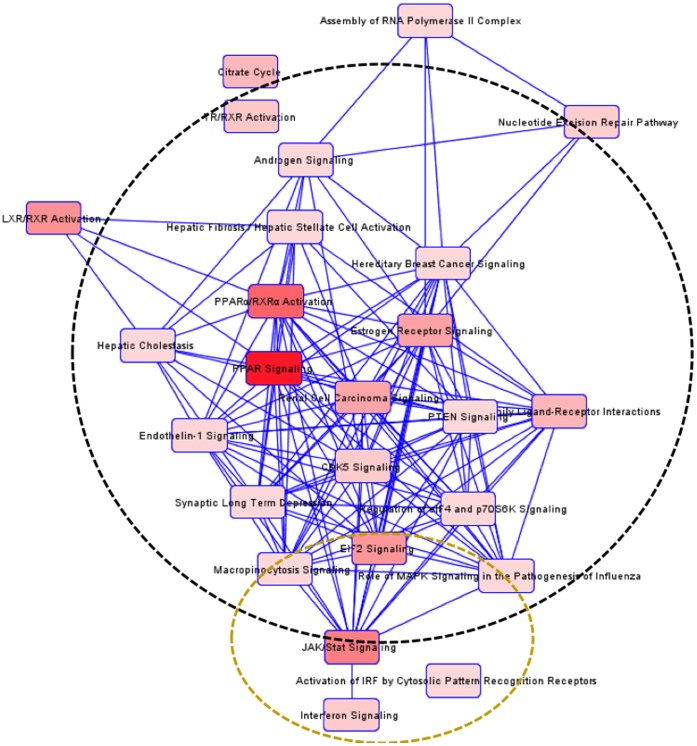
Detected interactions and clusters of enriched pathways in the HIP region in HIV-1 rats. All pathways identified by IPA software, including both significant and insignificant pathways, are interrelated and can be divided into two functional groups, indicated by circles.

**Figure 6:**
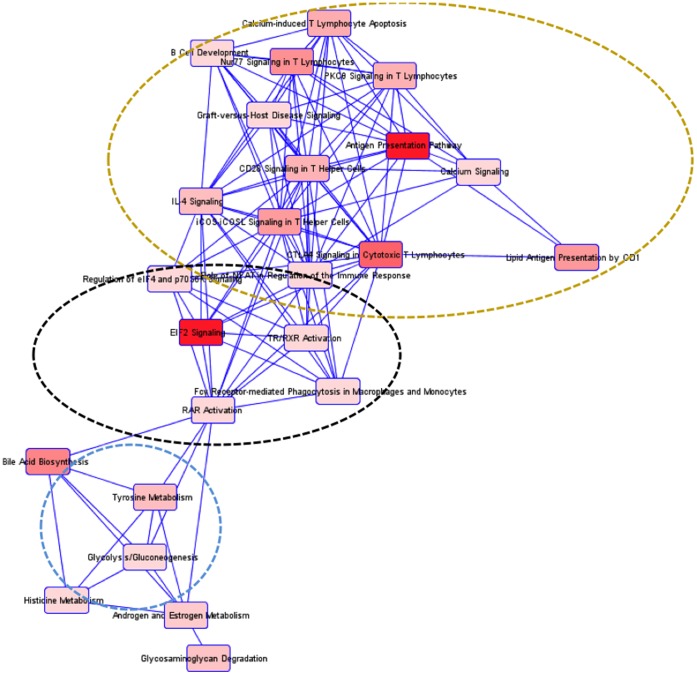
Detected interactions and clusters of enriched pathways in the STR region in HIV-1Tg rats. All pathways identified by IPA software, including both significant and insignificant pathways, are interrelated and can be divided into three functional groups, indicated by circles.

Among the pathways identified by IPA, including those altered at less stringent conditions, there was a clustering into immune response and neuronal survival in the three regions (Figs. 4, 5, 6). In the STR, there was a third cluster related to metabolism, which included tyrosine metabolism.

### Description of Important and Representative Genes Altered in the HIV-1Tg Rats

We considered important representative genes from the following three groups: immune-related genes (Table 2), neurotransmission-related genes (Table 3), and neuronal survival-related genes (Table 4). In the immune-related group (Table 2), mRNA expression of six cytokines and their receptors was significantly down-regulated in the HIV-1Tg rats, with chemokine ligand 2 (*Ccl2*, p = 0.0001), IK cytokine (*Ik,* p = 0.003), and lymphotoxin beta (*Ltb*, p = 0.0029) in the PFC, interleukin 1 receptor accessory protein (*Il1rap*, p = 0.0006) in the HIP, and chemokine ligand 6 (*Ccl6*, p = 0.0022) and chemokine receptor 10 (*Ccr10*, p = 0.0034) in the STR.

**Table 2:** Transcriptional changes in immune-related genes.

		PFC				HIP				STR			
Gene symbol	Gene name	FC ± STD	P-value	FDR	FC ± STD		P-value	FDR	FC ± STD		P-value	FDR	
**Cytokines and receptors**													
*Ccl2*	chemokine ligand 2	0.53±0.22***	0.0001	0.03	0.80±0.45		0.97	0.99	0.84±0.24		0.44	0.8	
*Ccl6*	chemokine ligand 6	0.98±0.16	0.89	0.97	0.56±0.51		0.0568	0.46	0.51±0.39**		0.0022	0.16	
*Ik*	IK cytokine, down-regulator ofHLA II	0.77±0.09**	0.003	0.17	1.00±0.10		0.99	1.00	0.93±0.11		0.46	0.8	
*Ltb*	lymphotoxin beta (TNFsuperfamily, member 3)	0.66±0.18**	0.0029	0.18	0.80±0.23		0.24	0.69	1.02±0.14		0.90	0.98	
*Ccr10*	chemokine receptor 10	1.10±0.07	0.22	0.63	0.99±0.26		0.97	0.99	0.75±0.10**		0.0034	0.18	
*Il1rap*	interleukin 1 receptor accessory protein	0.92±0.09	0.35	0.74	0.67±0.15***		0.0006	0.14	1.05±0.07		0.53	0.84	
**Kinases and enzymes**													
*Akt2*	v-akt murine thymoma viral oncogene homolog 2	1.47±0.24	0.28	0.69	0.95±0.25		0.81	0.96	0.26±0.40***		6.2E−09	1E−05	
*Chuk*	conserved helix-loop-helix ubiquitous kinase	0.61±0.17**	0.001	0.12	0.92±0.11		0.35	0.78	1.17±0.14		0.34	0.74	
*Irak4*	interleukin-1 receptor-associated kinase 4	0.77±0.09**	0.0028	0.17	0.88±0.06		0.0405	0.42	0.87±0.08		0.0653	0.44	
*Rras*	related RAS viral (r-ras) oncogene homolog	0.86±0.12	0.17	0.58	0.76±0.08***		0.0001	0.07	0.96±0.08		0.61	0.88	
**Transcription regulators**													
*Fos*	FBJ murine osteosarcoma viral oncogene homolog	0.49±0.42**	0.0025	0.17	0.62±0.30		0.0155	0.34	0.95±0.18		0.75	0.93	
*Hivep2*	human immunodeficiency virus typeI enhancer bindingprotein 2	1.03±0.06	0.70	0.91	1.77±0.07***		0.0007	0.16	1.15±0.09		0.20	0.64	
*Irf5*	interferon regulatory factor 5	1.64±0.09**	0.0049	0.20	0.72±0.21		0.0387	0.40	0.87±0.30		0.46	0.8	
*Irf7*	interferon regulatory factor 7	2.12±0.08***	0.0001	0.05	2.31±0.06***		3.60E−05	0.04	2.85±0.06***		9.10E−06	0.01	
*Litaf*	lipopolysaccharide-induced TNF factor	0.69±0.16**	0.0027	0.17	0.97±0.42		0.94	0.99	0.81±0.20		0.22	0.66	
*Pbxip1*	pre-B-cell leukemia homeobox interacting protein 1	0.94±0.13	0.64	0.89	1.08±0.17		0.66	0.91	0.48±0.45**		0.0026	0.17	
*Pias2*	protein inhibitor of activatedSTAT, 2	0.82±0.17	0.18	0.59	1.12±0.04		0.0429	0.42	1.53±0.08**		0.0014	0.15	
*Pias3*	protein inhibitor of activatedSTAT, 3	0.97±0.06	0.57	0.86	0.70±0.10***		0.0001	0.07	1.27±0.13		0.17	0.61	
*Stat6*	signal transducer and activator of transcription 6	0.64±0.17**	0.0014	0.13	1.22±0.11		0.22	0.67	0.89±0.12		0.32	0.73	

**Table 3:** Transcriptional changes in neurotranmission-related genes.

		PFC			HIP			STR		
Gene symbol	Gene name	FC ± STD	P-value	FDR	FC ± STD	P-value	FDR	FC ± STD	P-value	FDR
**Receptors and ion channles**										
*Clcn6*	chloride channel 6	1.02±0.07	0.73	0.92	1.39±0.07**	0.0027	0.23	1.07±0.06	0.33	0.74
*Hcrtr2*	hypocretin receptor 2	1.08±0.21	0.72	0.92	0.60±0.19**	0.0017	0.21	1.05±0.16	0.78	0.94
*Kcne4*	potassium voltage-gated channel, Isk-related family, member 4	0.61±0.24**	0.0031	0.18	0.78±0.13	0.0303	0.4	0.99±0.14	0.92	0.98
*Kcnip1*	Kv channel interacting protein 1	1.52±0.14	0.0726	0.44	1.49±0.08**	0.0023	0.22	1.73±0.10**	0.0037	0.18
*Grid1*	glutamate receptor, ionotropic, delta 1	1.27±0.06**	0.0041	0.20	1.17±0.07	0.0718	0.49	1.29±0.06**	0.0046	0.20
**Transporters**										
*Rtp4*	receptor transporter protein 4	1.49±0.07**	0.0043	0.20	1.70±0.07***	0.0007	0.15	1.29±0.07	0.0161	0.29
*Slc1a6*	high affinity aspartate/glutamate transporter	2.42±0.09***	0.0008	0.11	0.94±0.29	0.82	0.96	1.09±0.11	0.46	0.81
*Slc1a7*	glutamate transporter, member 7				0.54±0.33**	0.0031	0.24	0.78±0.36	0.38	0.77
**Enzymes and kinases**										
*Camk1*	calcium/calmodulin-dependent protein kinase I	0.93±0.06	0.22	0.63	1.00±0.22	1.00	1	0.53±0.26***	0.0008	0.14
*Camk2b*	calcium/calmodulin-dependent protein kinase II beta	1.25±0.05**	0.0024	0.17	1.12±0.09	0.28	0.72	1.19±0.11	0.21	0.64
*Gnal*	guanine nucleotide binding protein, alpha activating activity polypeptide, olfactory type	1.01±0.09	0.91	0.98	0.44±0.52**	0.0028	0.23	1.19±0.20	0.45	0.81
*Plcl2*	inactive phospholipase C-like protein 2	0.40±0.48***	0.0001	0.05	0.89±0.14	0.34	0.76	0.86±0.29	0.53	0.85
*Ppm1l*	protein phosphatase 1L	1.22±0.04**	0.0014	0.13	1.09±0.06	0.22	0.68	1.14±0.10	0.26	0.69
*Ppp1r14a*	Protein phosphatase 1 regulatory subunit 14A	0.75±0.11**	0.0030	0.18	0.85±0.21	0.37	0.78	0.65±0.20	0.0094	0.25
*Ppp2ca*	Serine/threonine-protein phosphatase 2A catalyticsubunit alpha isoform	1.76±0.08**	0.0012	0.13	0.94±0.04	0.09	0.52	1.14±0.11	0.34	0.74

**Table 4:** Transcriptional changes in genes related to neuronal survival.

		PFC			HIP			STR		
Gene symbol	Gene name	FC ± STD	P-value	FDR	FC ± STD	P-value	FDR	FC ± STD	P-value	FDR
**Myelin proteins**										
*Cldn1*	claudin 1	0.68±0.63	0.35	0.74	0.97±0.37	0.93	0.99	0.10±7.00**	0.0019	0.15
*Mag*	myelin associated glycoprotein	0.73±0.13**	0.0032	0.18	0.76±0.34	0.30	0.74	0.94±0.20	0.73	0.93
*Opalin*	oligodendrocytic myelin paranodal and inner loop protein	0.69±0.27	0.0497	0.40	0.96±0.21	0.85	0.97	0.43±0.57**	0.0036	0.18
**Growth factors and receptors**										
*Fgf13*	fibroblast growth factor 13	0.72±0.13**	0.0017	0.15	0.92±0.09	0.33	0.76	0.94±0.16	0.68	0.91
*Fgf9*	fibroblast growth factor 9 (glia-activating factor)	0.38±0.72**	0.0043	0.20	1.08±0.20	0.73	0.93	1.04±0.27	0.88	0.97
*Flt1*	fms-related tyrosine kinase 1	0.97±0.10	0.75	0.93	1.44±0.07**	0.0028	0.23	1.06±0.08	0.47	0.82
*HDGF_RAT*	hepatoma-derived growth factor	0.82±0.30	0.46	0.80	0.79±0.44	0.51	0.85	0.50±0.44**	0.0036	0.18
*Pdgfb*	platelet-derived growth factor beta polypeptide	0.84±0.20	0.34	0.74	1.59±0.08**	0.0045	0.26	1.02±0.18	0.93	0.98
*Wnt5a*	wingless-type MMTV integration site family, 5A	0.56±0.31**	0.0032	0.18	0.98±0.17	0.89	0.97	1.09±0.12	0.50	0.83
*Wnt5b*	wingless-type MMTV integration site family, 5B	0.69±0.15**	0.0024	0.17	1.06±0.10	0.63	0.90	1.19±0.13	0.27	0.69
*Grb14*	growth factor receptor-bound protein 14	0.74±0.11**	0.0013	0.13	0.86±0.07	0.0188	0.35	0.85±0.13	0.17	0.61
*Tbrg1*	transforming growth factor beta regulator 1	0.94±0.20	0.76	0.93	0.73±0.11**	0.0011	0.19	1.17±0.07	0.10	0.51
*Ephb1*	EPH receptor B1	0.88±0.03	0.0002	0.05	1.05±0.07	0.48	0.84	0.50±0.41**	0.0037	0.18
*Insr*	insulin receptor	1.14±0.14	0.41	0.78	1.41±0.06***	0.0004	0.14	0.97±0.07	0.67	0.91
**Translation regulators**										
*Eif2b5*	eukaryotic translation initiation factor 2B, subunit 5 epsilon, 82kDa	0.94±0.42	0.87	0.96	0.38±0.76	0.0191	0.35	0.14±0.49***	2.1E−15	0.00
*Eif2c1*	eukaryotic translation initiation factor 2C, 1	1.07±0.10	0.54	0.84	0.95±0.17	0.76	0.94	1.22±0.04**	0.0027	0.17
*Eif2c2*	eukaryotic translation initiation factor 2C, 2	1.24±0.09	0.0683	0.44	1.48±0.07**	0.002	0.22	1.08±0.13	0.61	0.88
*Eif3c*	eukaryotic translation initiation factor 3c	0.47±0.61	0.0178	0.30	0.40±0.64**	0.0036	0.24	0.91±0.05	0.0346	0.37
*Eif4a1*	eukaryotic translation initiation factor 4A1	0.67±0.14***	0.0004	0.08	0.88±0.12	0.24	0.69	0.76±0.13	0.0143	0.29
*Npm1*	nucleophosmin	0.29±0.62***	4.9E−07	0.00	0.43±0.18***	9.9E−10	0.00	0.43±0.22***	1.48E−08	0.00
*Rpl37*	ribosomal protein L37	0.73±0.10***	0.00086	0.11	0.66±0.32	0.11	0.56	1.43±0.09	0.014474	0.29
*Rps8*	ribosomal protein S8	0.74±0.10***	0.00085	0.11	1.10±0.07	0.27	0.72	0.97±0.07	0.67	0.91
*RL39_RAT*	ribosomal protein L39	0.89±0.04	0.00244	0.17	0.76±0.10**	0.00162	0.21	1.11±0.05	0.071592	0.45
*Rps24*	ribosomal protein S24	0.74±0.24	0.09811	0.49	0.71±0.13**	0.00178	0.21	0.89±0.14	0.32	0.73
*Rpl13*	ribosomal protein L13	0.82±0.21	0.25	0.67	1.06±0.11	0.62	0.90	2.08±0.10**	0.002067	0.15
*Rpl35*	ribosomal protein L35	1.82±0.15	0.05657	0.41	0.91±0.33	0.76	0.94	2.80±0.03***	3.44E−09	0.00
*Rps19*	ribosomal protein S19	0.86±0.11	0.15	0.55	0.86±0.17	0.33	0.76	1.66±0.08**	0.00229	0.16

The HIV-1Tg rat also showed decreased expression of immune-related kinases and enzymes, specifically conserved helix-loop-helix ubiquitous kinase (*Chuk*; p = 0.001) and interleukin-1 receptor-associated kinase 4 (*Irak4,* p = 0.0028) in the PFC, r-ras oncogene homolog (*Rras*; p = 0.0001) in the HIP, and v-akt murine thymoma viral oncogene homolog 2 (*Akt2*; p = 6.2 × 10^-9^) in the STR.

Nine transcription regulators related to immune responses were different in the HIV-1Tg rat. The expression of interferon regulatory factor 7 (*Irf7*) was significantly increased in all the three brain regions (PFC: fold change [FC] = 2.12±0.08; p = 0.0001; HIP: FC = 2.31±0.06; p = 3.6 × 10^-5^; STR: FC = 2.85±0.06; p = 9.1 × 10^-6^). The rest of the genes were changed in only one brain region, with down-regulation of FBJ murine osteosarcoma viral oncogene homolog (*Fos*; p = 0.0025), lipopolysaccharide-induced TNF factor (*Litaf*; p = 0.0027), and signal transducer and activator of transcription 6 (*Stat6*; p = 0.0014) in the PFC, protein inhibitor of activated STAT-3 (*Pias3*; p = 0.0001) in the HIP, and pre-B-cell leukemia homeobox interacting protein 1 (*Pbxip1*; p = 0.0026) in the STR; and up-regulation of interferon regulatory factor 5 (*Irf5*; p = 0.0049*)* in the PFC, human immunodeficiency virus type I enhancer binding protein 2 (*Hivep2*; p = 0.0007) in the HIP, and protein inhibitor of activated STAT 2 (*Pias2*; p = 0.0014) in the STR.

As shown in Table 3, neurotransmission-related genes could be further subgrouped into a) receptors and ion channels, b) transporters, and c) enzymes and kinases. Five genes encoding receptors and ion channels were significantly changed in the HIV-1Tg rat, with down-regulation of potassium voltage-gated channel, isk-related family, member 4 (*Kcne4*; p = 0.0031) in the PFC and hypocretin receptor 2 (*Hcrtr2*; p = 0.0017) in the HIP and up-regulation of glutamate receptor, ionotropic, delta 1 (*Grid1*; p = 0.0041) in the PFC, chloride channel 6 (*Clcn6*; p = 0.0027) and Kv channel interacting protein 1 (*Kcnip1*; p = 0.0023) in the HIP, and *Kcnip1* (p = 0.0037) and *Grid1* (p = 0.0046) in the STR.

Transporters, including receptor transporter protein 4 (*Rtp4*; p = 0.0043) and high-affinity aspartate/glutamate transporter (*Slc1a6*; p = 0.0008), were up-regulated in the PFC. *Rtp4* also was up-regulated in the HIP (p = 0.0007), whereas glutamate transporter member 7 (*Slc1a7)* was down-regulated in this region (p = 0.0031). Calcium/calmodulin-dependent protein kinase2b (*Camk2b*) was up-regulated in the PFC (p = 0.0024), whereas *Camk1* was down-regulated in the STR (p = 0.0008). Guanine nucleotide binding protein (*Gnal*) was down-regulated only in the HIP (p = 0.0028). Phospholipases were altered only in the PFC, with inactive phospholipase C-like protein 2 (*Plcl2*; p = 0.0001) and protein phosphatase 1 regulatory subunit 14A (*Ppp1r14*; p = 0.003) being down-regulated, and protein phosphatase 1L (*Ppm1l*; p = 0.0014) and serine/threonine-protein phosphatase 2A catalytic subunit alpha isoform (*Ppp2ca*; p = 0.0014) being up-regulated.

The neuronal survival-related genes were subgrouped (Table 4) into a) myelin proteins, b) growth factors and receptors, and c) translation regulators. There was down-regulation of myelin-associated glycoprotein (*Mag*; p = 0.0032) in the PFC and claudin 1 (*Cldn1*; p = 0.0019*)* and oligodendrocytic myelin paranodal and inner loop protein (*Opalin*; p = 0.0036) in the STR. Eleven genes encoding growth factors and their receptors were altered in the HIV-1Tg rats, with down-regulation of fibroblast growth factor 13 (*Fgf13*; p = 0.0017), fibroblast growth factor 9 (*Fgf9*; p = 0.0043), wingless-type MMTV integration site family 5A (*Wnt5a*, p = 0.0032), wingless-type MMTV integration site family 5B (*Wnt5b*; p = 0.0024), and growth factor receptor-bound protein 14 (*Grb14*; p = 0.0013) in the PFC; transforming growth factor beta regulator 1 (*Tbrg1;* p = 0.0011) in the HIP; and hepatoma-derived growth factor (*HDGF_RAT*; p = 0.0036) and EPH receptor B1 *(Ephb1*; p = 0.0037) in the STR, and up-regulation of fms-related tyrosine kinase 1 (*Flt1*; p = 0.0028), platelet-derived growth factor beta polypeptide (*Pdgfb*; p = 0.0045), and insulin receptor (*Insr,* p = 0.0004) in the HIP.

Translation initiation factors (Eif) were also changed in the HIV-1Tg rat, with down-regulation of *Eif4a1* in the PFC (p = 0.0004), *Eif3c* in the HIP (p = 0.0036), and *Eif2b5* in the STR (p = 2.16 × 10^-15^), and up-regulation of *Eif2c2* in the HIP (p = 0.002) and *Eif2c1* in the STR (p = 0.0027).

Moreover, various ribosomal proteins (Rp) were changed in the HIV-Tg rat in a brain region-dependent manner, with down-regulation of *Rpl37* (p = 0.00086) and *Rps8* (p = 0.00085) in the PFC, and *Rl39_Rat* (p = 0.00162) and *Rsp24* (p = 0.00178) in the HIP, but up-regulation of *Rpl13* (p = 0.0021), *Rpl35* (p = 3.44E−09), and *Rps19* (p = 0.00229) in the STR. Nucleophosmin (*Npm1*), a gene implicated in ribosomal transport, was down-regulated in all three brain regions (PFC, FC = 0.29±0.62; HIP, FC = 0.43±0.18; STR, FC = 0.43±0.22; p<5.0 × 10^-7^ for all brain regions).

### qRT-PCR Confirmation on Representative Genes

**Figure 7:**
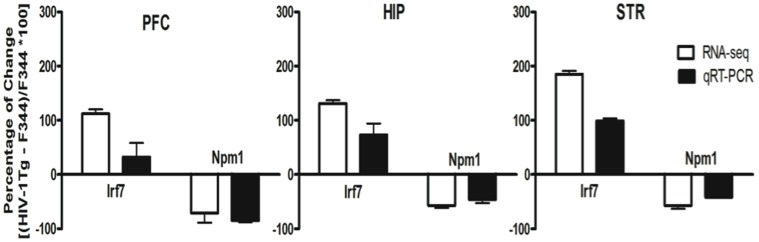
qRT-PCR confirmation on the expression of interferon regulatory factor 7 (*Irf7*) and Nucleophosmin (*Npm1*) in the PFC, HIP and STR. Both RNA-seq analysis and qRT-PCR showed that *Irf7* was upregulated while *Npm1* was downregulated in all the three brain regions of HIV-1Tg rats as compared to F344 rats. The expression level of a gene of interest was shown in the figure as percentage of [(HIV-1Tg – F344)/F344*100] in each brain region for both approaches.

We selectively examined the expression of *Irf7* and *Npm1*, the only two genes showing significant strain differences across all the three brain regions (Tables 2 and 3). Consistent with the findings from the RNA-sequencing analysis, qRT-PCR showed significant alterations in the expression of these two genes between HIV-1Tg and F344 rats in all the three brain regions (Fig. 7).

## Discussion

**Figure 8:**
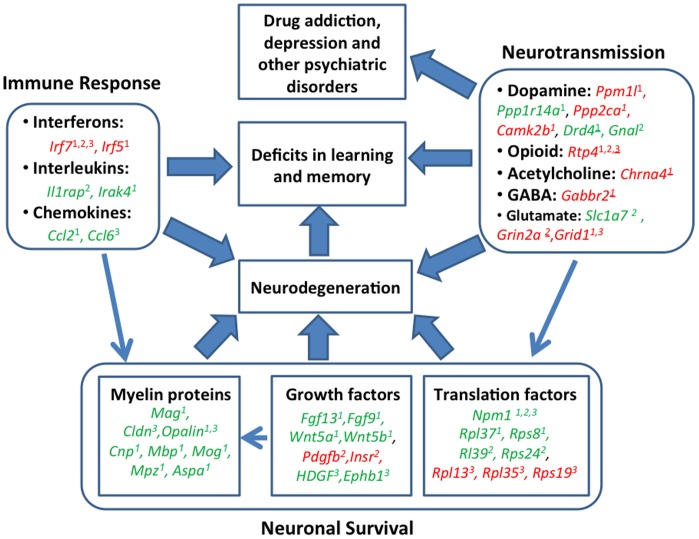
Summary of represented genes and enriched biochemical pathways related to neurobehavioral deficits in the HIV-1Tg rat. Red represents up-regulation, whereas green represents down-regulation in mRNA expression in the PFC^1^, HIP^2^, and STR^3^ of HIV-1Tg rats. Numbers with strikethroughs reflect marginal significance (0.005<p<0.05; see Table S1). Abbreviations: Aspa = aspartoacylase; Camk2b = calcium/calmodulin-dependent protein kinase II beta; Ccl2 =  chemokine ligand 2; Ccl6 =  chemokine ligand 6; Chrna4 =  neuronal acetylcholine receptor subunit alpha-4; Cldn = Claudin 1; Cnp = 2,3-cyclic-nucleotide 3-phosphodiesterase; Drd4 =  D(4) dopamine receptor; Ephb1 =  EPH receptor B1; Fgf9 =  fibroblast growth factor 9; Fgf13 =  fibroblast growth factor 13; Gabbr2, Gnal = guanine nucleotide binding protein, alpha activating activity polypeptide; Grid1 =  glutamate receptor, ionotropic, delta1; Grin2a = NMDA receptor subunit epsilon-1; HDGF = hepatoma-derived growth factor; Il1rap = interleukin 1 receptor accessory protein; Insr = insulin receptor; Irak4, interleukin-1 receptor-associated kinase 4; Irf5 =  interferon regulatory factor 5; Irf7 =  interferon regulatory factor 7; Mag = myelin-associated glycoprotein; Mbp = myelin-associated glycoprotein; Mog = myelin-oligodendrocyte glycoprotein; Mpz = myelin protein P0; Npm1 =  nucleophosmin; Opalin = oligodendrocytic myelin paranodal and inner loop protein; PDGFb = platelet-derived growth factor beta polypeptide; Ppm1l = protein phosphatase 1L; Ppp1r14a = protein phosphatase 1 regulatory subunit 14A; Ppp2ca = serine/threonine-protein phosphatase 2A catalytic subunit alpha isoform; Rl39 =  ribosomal protein L39; Rpl13 =  ribosomal protein L13; Rpl35 =  ribosomal protein L35; Rpl37 =  ribosomal protein L37; Rps8 =  ribosomal protein S8; Rps19 =  ribosomal protein S19; Rps24 =  ribosomal protein S24; Rtp4 =  receptor transporter protein 4; Slc1a7 =  glutamate transporter, member 7; Wnt5a = wingless-type MMTV integration site family, 5A; Wnt5b = wingless-type MMTV integration site family, 5B.

Although HIV-1Tg rats have been used as a model to study neurobehavioral deficits in learning and memory and drug addiction in HIV-1-positive patients, the complete gene expression profile in the CNS of this animal model is undetermined. The present study, therefore, evaluated “exon-wide” gene expression in the brain of the HIV-1Tg rat. In contrast to previous studies evaluating the expression of limited numbers of genes in general brain homogenates [46], [47], we examined gene expression in specific brain regions important in motor and motivation control, learning, and memory. Our study showed that viral proteins without viral reproduction primarily altered the expression of genes and pathways related to immune responses, neurotransmission, and neuronal survival, although some overlaps exist. The alteration in gene expression depended highly on the brain region examined. Thus, the observed abnormal gene expression points to the mechanisms underlying HIV-1-induced neurobehavioral deficits as discussed below and summarized in Figure 8.

### Immune-related Pathways and Genes

Consistent with previous reports [32], [47], many pathways and genes directly related to immune responses were found to be altered in the HIV-1Tg rat. We found cytokine-related genes, including those for interferon, interleukins, and chemokines, and pathways were altered in the HIV-1Tg animals. Interferon-related pathways, such as Toll-like receptor signaling, interferon signaling, and IL-4 signaling, were altered in the PFC, HIP, and STR, respectively. Evaluation of the genes involved in these pathways revealed that interferon regulatory factor 7 (*Irf7*) was significantly up-regulated in all three brain regions. Moreover, Irf5, which can form heterodimers with Irf7 to modulate target gene expression [48], was up-regulated in the PFC. Given that Irf7 is the master regulator of type I interferon production and is involved in oncogenesis and apoptosis [49], alteration of that pathway and up-regulation of *Irf7* suggest disturbances of the innate immune response and neuronal survival in the HIV-1Tg rat.

Interleukins and their receptors were generally unchanged in the HIV-1Tg rat, although the down-regulation of interleukins 4 and 13 receptors was marginally significant in the PFC (Table S1). However, *Il1rap* and *Irak4*, which regulate the function of interleukin-1 receptor [50], [51], were significantly down-regulated in the HIP and PFC, respectively. Given that the interleukin-1 receptor plays an important role in hippocampus-dependent spatial memory [52], [53], the down-regulation of genes in interleukin-1 signaling may contribute to the decreased performance in the Morris water maze test observed in HIV-1Tg rats [30], [31].

We also detected significant down-regulation of chemokines *Ccl2* and *Ccl6* in the PFC and STR, respectively, in the HIV-1Tg rats. Furthermore, the down-regulation of *Ccl2* in the HIP and Ccl2 receptor in the PFC was marginally significant. CCL2 has been reported to support cortical neuron survival and plays a neuroprotective role under toxin challenge [54]. The protein also induces neuronal differentiation and oligodendrocyte maturation [55]. Therefore, the down-regulation of this gene may contribute to cortical neurodegeneration and myelin deficits. This hypothesis is supported by the observation of decreased expression of myelin-related genes, especially in the PFC (Table 3, Table S1). Interestingly, clinical studies have shown that increased CCL2 is associated with HAND by facilitating HIV-1 viral replication and movement across the blood-brain barrier [56], [57]. This discrepancy may reflect a difference between mRNA and protein or gene expression in specific brain regions versus whole brain. Because the HIV-1Tg rat does not have any viral replication or reproduction, the discrepancy also suggests that HAND, with controlled viral reproduction, has neuropathology distinct from that associated with active viral reproduction. On the other hand, the decreased expression of *Ccl6*, a chemokine which induces astrocyte migration, may reduce the reparability of damaged brain regions [58].

Thus, our finding of alterations in immune-related genes and pathways suggests not only an abnormal innate immune response, but also neurodegeneration in the CNS, which may contribute to the cognitive deficits observed in the HIV-1Tg rat.

### Neurotransmission-related Genes and Pathways

Our results also suggest that dopamine transmission is altered in the CNS of the HIV-1Tg rat. In the PFC, dopamine receptor signaling was significantly altered at the pathway level, which was reflected mainly by a change in the expression of protein phosphatases (*Ppm1l*, *ppp1r14a*, *Ppp2ca*). We also found decreased expression of dopamine receptor 4 (*Drd4*) in the PFC of HIV-1Tg rats. Drd4 regulates emotional memory by modulating calmodulin-dependent kinase II and subsequently affecting AMPA receptor-mediated excitatory synaptic transmission [59], [60]. Interestingly, we also found abnormal expression of calmodulin-dependent kinase II (*Camk2b*) in the PFC. These data suggest a deficit in glutamate transmission and memory related to emotion in the HIV-1Tg rat. In the STR, tyrosine metabolism, which is important in the synthesis of monoamines such as dopamine, norepinephrine, and epinephrine, was significantly altered at the pathway level. Although we did not detect any dopamine-related pathway changes in the HIP, Gnal, which links dopamine receptor 1 and adenylyl cyclase [61], was down-regulated in the HIP. This protein affects the responses to psychostimulants and is involved in many psychiatric disorders, such as major depression, schizophrenia, and bipolar disorder [62]–[64]. Together, alterations in dopamine-related genes and pathways suggest a molecular mechanism underlying the vulnerability of HIV-positive patients to drug addiction, depression, and other psychiatric disorders.

Although glutamate transmission was not significantly altered at the pathway level, we detected a few modified receptors in the HIV-1Tg rat. For example, Grid1, a subunit of the glutamate receptor, with polymorphisms associated with schizophrenia [65], was overexpressed in both the PFC and the STR of HIV-1Tg rats. Moreover, we discovered overexpression of the NMDA receptor (*Grin2a*) in the HIP in the HIV-1Tg rat. Because there is also down-regulation of glutamate transporter (*Slc1a7*) in this region, there could be an increased excitotoxicity that leads to neurodegeneration in the HIP of the HIV-1Tg rat.

In addition to the affected genes in the dopamine and glutamate systems, we detected abnormal expression of a few genes in other neurotransmitter systems that regulate responses to psychostimulants. First, *Rtp4*, a receptor transporter involved in opioid receptor heterodimer formation [66], was up-regulated in the PFC and HIP, and showed marginally significant up-regulation in the STR of the HIV-1Tg rats (p = 0.016). Second, the up-regulation of the GABAb receptor subunit (*Gabbr2*) in the PFC was marginally significant (p = 0.03). Polymorphism of *Gabbr2* is associated with nicotine and alcohol dependence [67], [68], and increased transcription of *Gabbr2* is observed in the PFC of nicotine-treated and alcohol-addicted animals [69]–[71]. Third, the up-regulation of the cholinergic receptor (*Chrna4*; p = 0.037) is marginally significant in the PFC of the HIV-1Tg rat. Given the involvement of these genes in drug addiction, altered expression could contribute to the abnormal behavioral responses to psychostimulants, including alcohol, nicotine, and morphine, in the HIV-1Tg rat [33], [34], [72].

### Neuronal Survival-related Pathways and Genes

Myelin is an important structure, supporting neuronal survival and function [73], [74]. Deficits in central myelin have been observed in the brains of HIV-1-positive patients [75], [76] and are resistant to anti-retroviral therapy. Myelin abnormality is, thus, suggested to contribute to neurocognitive changes defying HAART [77]. We observed a similar down-regulation of myelin genes in the HIV-1Tg rat, which included *Mag* in the PFC and *Opalin* and *Cldn1* in the STR. With a lower significance threshold, we observed more myelin-related genes with decreased expression in the PFC, which included 2, 3-cyclic-nucleotide 3-phosphodiesterase, myelin basic protein, myelin-oligodendrocyte glycoprotein, and aspartoacylase (Table S1). These alterations may lead to neurodegeneration in the CNS of the HIV-1Tg rat.

Growth factors regulate both myelin formation and neuronal survival. We observed distinct growth factor and receptor alterations in different brain regions. In the PFC, Wnt/β-catenin signaling was significantly altered. The HIV-1Tg rats showed decreased expression of *Wnt7a*, an endogeneous ligand for Wnt/β-catenin signaling [78], with marginal significance. *Wnt5a*, which also regulates β-catenin [79], showed significant down-regulation in the HIV-1Tg rats as well. Activation of the Wnt/β-catenin pathway induces myelin gene expression, supports neuronal survival [80], [81], and improves spatial memory [82]. Moreover, Fgf9 and Fgf13, the two fibroblast growth factors supporting neuronal survival and myelin gene expression [83]–[87], showed decreased expression in the PFC of HIV-1Tg rats. Therefore, the decreased expression of these genes may contribute to abnormal myelin gene expression in this brain region. Although these genes were not significantly altered in the STR, hepatoma-derived growth factor (*HDGF*) and EPH receptor (*Ephb1*), which have neuroprotective functions [88], [89], showed decreased expression in the STR of HIV-1Tg rats. In contrast to the PFC and STR, we found up-regulation of growth factors and receptors, including insulin receptor and platelet-derived growth factor (*Pdgfb*). Increased insulin receptor expression has been observed under pathological conditions, such as Alzheimer’s dementia and multiple sclerosis [90]–[92], and increased *Pdgfb* expression has also been observed in Alzheimer’s disease patients and simian human immunodeficiency virus-infected macaques with encephalitis [93], [94]. The up-regulation of these genes suggests disturbed protein homoeostasis and deficits in macrophage function in the HIP of the HIV-1Tg rat [91], [95].

We also found significant alteration in translation regulators, including the translation initiation factors and ribosomal proteins. The down-regulation of ribosomal proteins in both the PFC and HIP suggests down-regulation in translation activity in the HIV-1Tg rat. In contrast, a different set of ribosomal proteins was up-regulated in the STR. However, Npm1, important in nuclear export of ribosomal subunits [96], was down-regulated in all three brain regions, which suggests a general down-regulation of protein synthesis in the HIV-1Tg rat.

### Conclusions

Our “exon-wide” gene expression profile showed that genes and pathways related to immune responses, neurotransmission, and neuronal survival were altered in the CNS of the HIV-1Tg rat in a brain region-dependent manner. Further, these pathways are highly interconnected in that both immune responses and neurotransmission affect neuronal survival (Fig. 8). Alterations in these genes and pathways suggest directions for more thorough mechanistic studies on the neurobehavioral deficits observed in the HIV-1Tg rat. For example, abnormal gene expression in all three groups suggests neuronal degeneration in the CNS of the HIV-1Tg rat, which contributes to the deficits in learning and memory (Fig. 8). Moreover, changes in neurotransmission in the brain region important in emotion and motivation control could underlie the abnormal responses to psychostimulants as well as psychiatric disorders (Fig. 8). Further clarification of these mechanisms will help in the development of efficient therapies to treat HAART-resistant HAND, drug addiction, and other psychiatric disorders in HIV patients.

## Supporting Information

Table S1 **The genes showing alterations with marginal significance in HIV-1 Tg rats.**
(DOCX)
