# Supplementary material for: Transcriptome Sequencing of Gene Expression in the Brain of the HIV-1 Transgenic Rat
Source: PLoS One. 2013 Mar 25;8(3):e59582. doi: 10.1371/journal.pone.0059582 (PMC3607591; doi:10.1371/journal.pone.0059582)
Supplement: Table S1 — The genes showing alterations with marginal significance in HIV-1 Tg rats. (DOCX) [file pone.0059582.s001.docx]

**Table S1: The genes showing alterations with marginal significance in HIV-1 Tg rats**

| Region | Gene symbol | Gene name | FC ± STD | P-value | FDR |
| --- | --- | --- | --- | --- | --- |
| PFC | Aspa | Aspartoacylase | 0.74 ± 0.14 | 0.022 | 0.32 |
|  | Ccr2 | C-C chemokine receptor type 2 | 0.52 ± 0.48 | 0.027 | 0.34 |
|  | Chrna4 | Neuronal acetylcholine receptor subunit alpha-4 | 1.71 ± 0.10 | 0.005 | 0.21 |
|  | Drd4 | D(4) dopamine receptor | 0.58 ± 0.43 | 0.037 | 0.37 |
|  | Gabbr2 | Gamma-aminobutyric acid type B receptor subunit 2 | 1.31 ± 0.09 | 0.030 | 0.35 |
|  | Il13ra2 | Interleukin-13 receptor subunit alpha-2 | 0.66 ± 0.26 | 0.025 | 0.33 |
|  | Il4ra | Interleukin-4 receptor subunit alpha | 0.52 ± 0.41 | 0.007 | 0.22 |
|  | MBP_RAT | Myelin basic protein S | 0.76 ± 0.13 | 0.013 | 0.27 |
|  | Mog | Myelin-oligodendrocyte glycoprotein | 0.71 ± 0.20 | 0.027 | 0.34 |
|  | Mpz | Myelin protein P0 | 0.70 ± 0.21 | 0.024 | 0.33 |
| HIP | Gria3 | Glutamate receptor 3 | 1.34 ± 0.10 | 0.042 | 0.42 |
|  | Grik1 | Glutamate receptor, ionotropic kainate 1 | 1.39 ± 0.09 | 0.015 | 0.34 |
|  | Grin2a | Glutamate [NMDA] receptor subunit epsilon-1 | 1.33 ± 0.08 | 0.012 | 0.32 |
|  | Gabrr2 | Gamma-aminobutyric acid receptor subunit rho-2 | 1.55 ± 0.10 | 0.013 | 0.33 |

Note: Marginal significance was defined as 0.005 <p <0.05 with over 20% change in expression.
